# Identification of RHOBTB2 aberration as an independent prognostic indicator in acute myeloid leukemia

**DOI:** 10.18632/aging.203087

**Published:** 2021-06-01

**Authors:** Peng Liu, Qinghai Ma, Hanxiang Chen, Li Zhang, Xiaoning Zhang

**Affiliations:** 1 Department of Clinical Laboratory Medicine, The First Affiliated Hospital of Shandong First Medical University & Shandong Provincial Qianfoshan Hospital, Shandong Medicine and Health Key Laboratory of Laboratory Medicine, Jinan, Shandong Province, PR China

**Keywords:** acute myeloid leukemia, bioinformatics analysis, Rho-related BTB domain-containing protein 2 Gene, prognosis

## Abstract

Rho-related BTB domain (RhoBTB) proteins belong to Rho guanosine triphosphatases (GTPases). Their putative role implicated in carcinogenesis has been supported by accumulating evidence. However, their expression pattern and potential role in acute myeloid leukemia (AML) remain unclear.

We profiled RHOBTB mRNA expression via the Gene Expression Profiling Interactive Analysis 2 (GEPIA2) database. Survival analysis was conducted with GEPIA2 and UALCAN. Univariate and multivariate Cox regression analyses were performed to validate RHOBTB genes as independent prognostic indicators in the LAML cohort from The Cancer Genome Atlas (TCGA). Data regarding expression in different subtypes and relationships with common disease-related genes were retrieved from UALCAN. Co-expressed genes were screened out and subsequently subjected to functional enrichment analysis.

We observed aberrant transcription levels of RHOBTB genes in AML patients. RHOBTB2 was identified as a prognostic candidate for overall survival (OS), independent of prognosis-related clinical factors and genetic abnormalities. Moreover, RHOBTB2 expression was increased in non-acute promyelocytic leukemia (APL) subtypes, patients without FLT3 mutation and PML/RAR fusion, and imparted a positive correlation with the expression of FLT3, FHL1, and RUNXs. Co-expressed genes of RHOBTB2 were enriched in functional pathways in AML.

Our findings suggest that RHOBTB2 might be a novel biomarker and independent prognostic indicator in AML and provide insights into the leukemogenesis and molecular network of AML.

## INTRODUCTION

Acute myeloid leukemia (AML) is the most common form of acute leukemia in adults, and the incidence and mortality risks increase with age [1, 2]. As a heterogeneous disease of the blood system, AML is characterized by differentiation arrest and malignant clonal expansion of myeloid lineage blasts. Many oncogene activating mutations and cytogenetic abnormalities in AML, such as core-binding factor (CBF), retinoic acid receptor-α (RAR-α), FLT3, RAS, p53, WNT, nucleophosmin (NPM1), and CEPBA^*double*^, are associated with high-risk clinical characteristics and adverse prognosis [3–5]. The complex genetic background substantially impacts risk stratification, treatment responses, and prognosis prediction. Hence, it is urgent to authenticate potential and independent biomarkers involved in diagnosis, treatment, and prognosis of patients with AML.

The RhoBTB subfamily, represented in mammals by three isoforms, RhoBTB1, RhoBTB2, and RhoBTB3, was recognized in the lower eukaryote *Dictyostelium discoideum*, and thus became the *de novo* addition to Rho-related proteins [6]. As atypical members of small guanosine triphosphatases (GTPases), RhoBTB proteins possess a salient architecture: a proline-rich region follows the GTPase domain, a tandem of two BTB (broad complex, tramtrack, and bric-a-brac) domains, and a carboxyl-terminal BTB and C-terminal Kelch (BACK) domain [7]. RHOBTB genes have been identified as tumor suppressors and are reduced or abolished in diverse solid tumors. RHOBTB2 (also called deleted in breast cancer 2 (DBC2)) has been reported as a gene homozygously deleted in breast cancer samples. It exerts a tumor-suppressive function by inhibiting cancer cell proliferation, migration, and invasiveness [8, 9]. Silenced RHOBTB2 expression has been further observed in lung, bladder, bone, and gastric cancer [10–14]. RHOBTB1 is heterozygously deleted in head and neck squamous cell carcinomas (HNSCCs), and its expression is silenced in colon cancer by miR-31 [15]. RHOBTB3 is significantly decreased in renal carcinoma and acts as a tumor suppressor by promoting ubiquitination and degradation of HIFa [16]. To date, little is known about the expression profiles of RHOBTB genes and their relationships with clinicopathological features and prognosis in leukemia. Here, we applied bioinformatics analyses to determine the expression of RHOBTB genes in AML patients based on large-scale gene expressions in copy numbers published online and validate RHOBTB2 as an independent prognostic indicator and a tumor biomarker.

## RESULTS

### Transcriptional levels of RHOBTB genes in patients with AML

**Figure 1:**
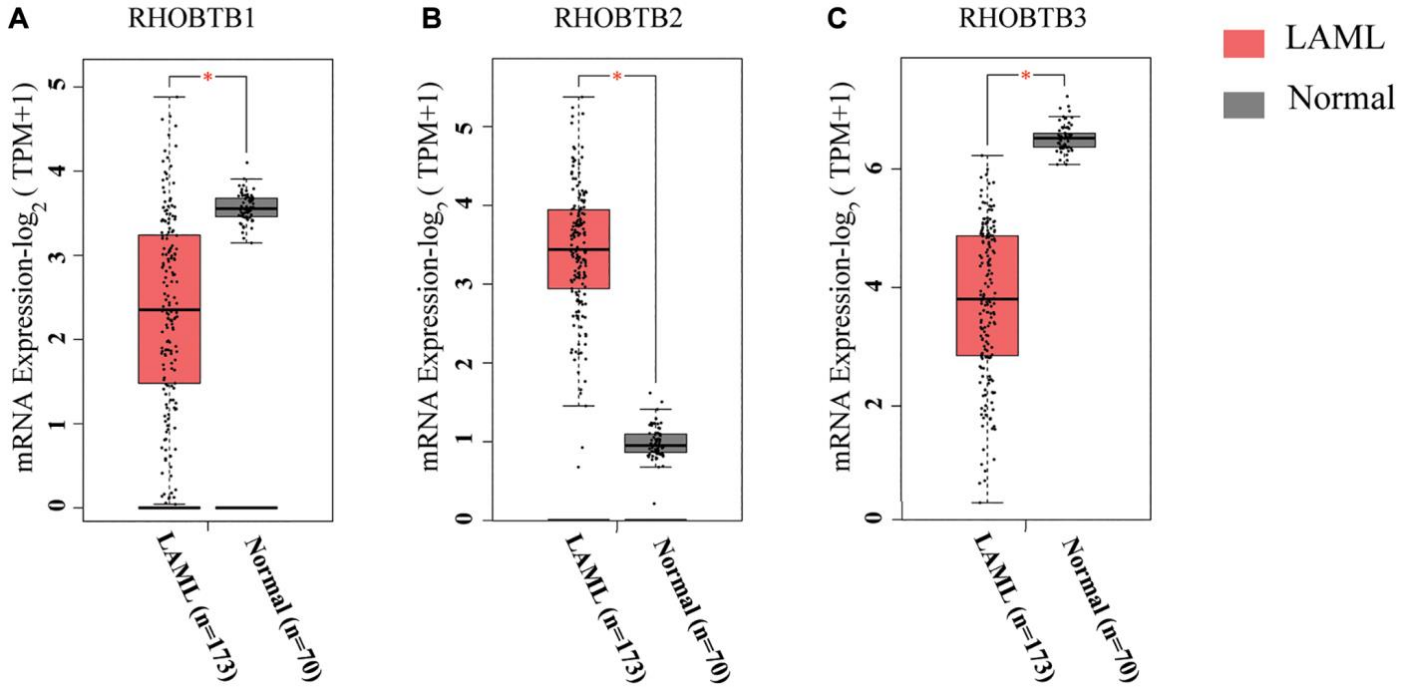
**RHOBTB mRNA expression levels in patients with AML (GEPIA2).** Box plots show the expression profiles of RHOBTB1 (**A**), RHOBTB2 (**B**), and RHOBTB3 (**C**) in bone marrow samples of patients in the TCGA-AML cohort (*n* = 173) compared to those in normal matched samples (*n* = 70) from GTEx. The transcriptional levels were log-normalized by the log_2_(TPM+1) method. A *t*-test was used to compare the differences in expression between tumor and normal tissues. ^*^P < 0.05.

The RHOBTB genes comprise three members, RHOBTB1, RHOBTB2, and RHOBTB3, in mammalian cells. We compared the transcriptional levels of RHOBTB genes in the bone marrow of AML patients (TCGA-LAML, *n* = 173) with those in normal samples (GTEx, *n* = 70) through Gene Expression Profiling Interactive Analysis 2 (GEPIA2) (Figure 1). Gene expression analysis using box plots indicated that the transcriptional levels of RHOBTB1 and RHOBTB3 were decreased (*P <* 0.05, Figure 1A and 1C), while that of RHOBTB2 was significantly increased (*P <* 0.05, Figure 1B). Consistently, three datasets indicated increased RHOBTB2 expression; six datasets and five datasets showed reduced RHOBTB1 and RHOBTB3 expression, respectively, in leukemia compared to normal samples in the ONCOMINE database (Supplementary Figure 1). Downregulation of RHOBTB1 and RHOBTB3 has been reported in various types of tumors, and this pattern was confirmed in AML. RHOBTB2 showed notably divergent expression patterns between AML and other tumor types, which led us to further explore the underlying clinical significance.

### Prognostic values of RHOBTB genes in patients with AML

**Figure 2:**
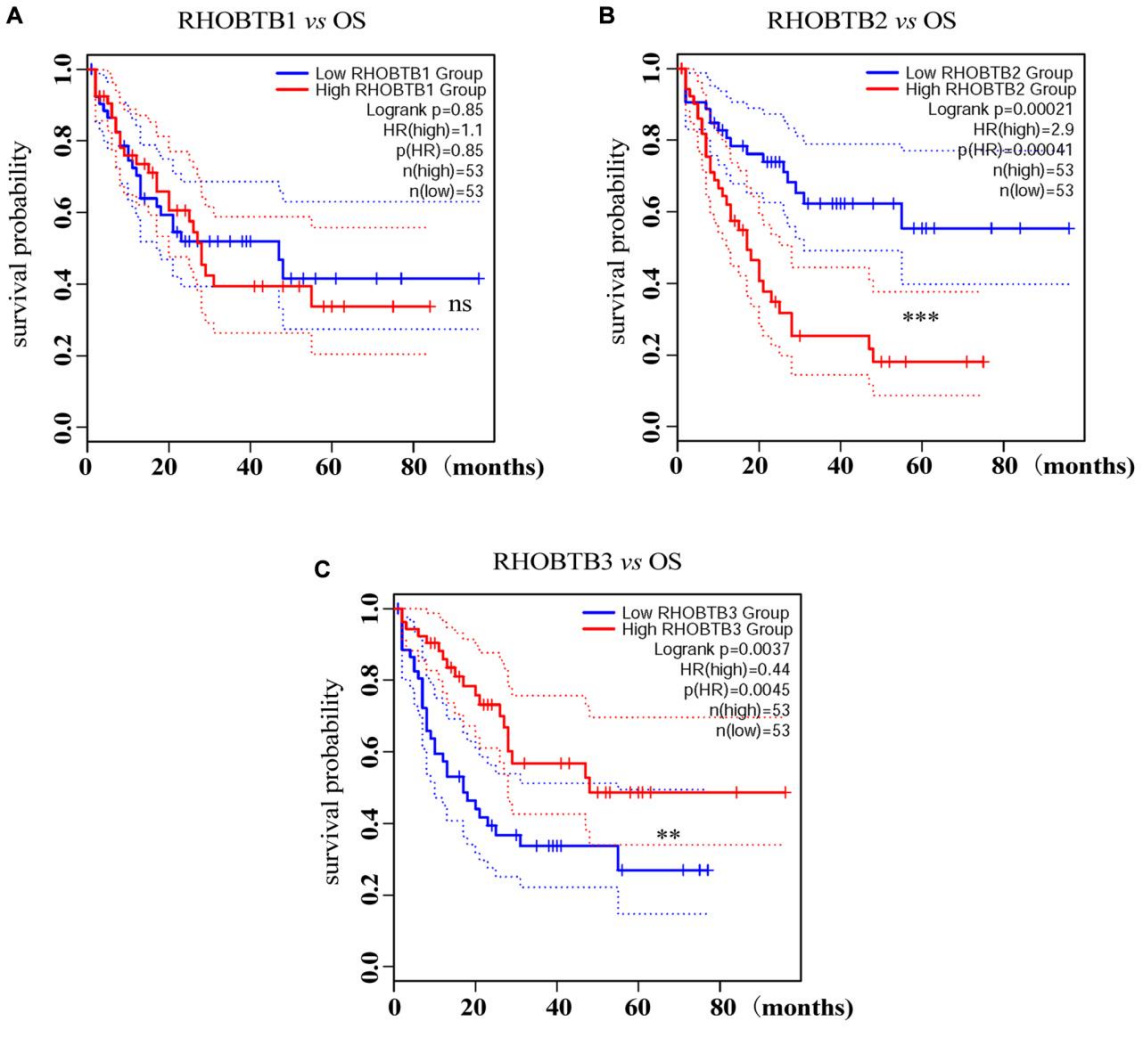
**Prognostic analysis of RHOBTB gene expression in AML patients (GEPIA2).** Survival analysis was performed based on the mRNA expression levels of RHOBTB1 (**A**), RHOBTB2 (**B**), and RHOBTB3 (**C**) and survival status in the TCGA-AML cohort (106 patients were analyzed) via GEPIA2. Kaplan-Meier (KM) curves were plotted with *P*-values and HRs by log-rank tests and Cox regression models. Dotted lines indicate the 95% CI. Gene expression levels were dichotomized, generating a high expression group (solid red line) and a low expression group (solid blue line), based on the median expression level of each gene as the cut-off value. OS, overall survival. HR, hazard ratio. CI, confidence interval. ^**^P < 0.01. ^***^P < 0.001. ns, not significant.

**Figure 3:**
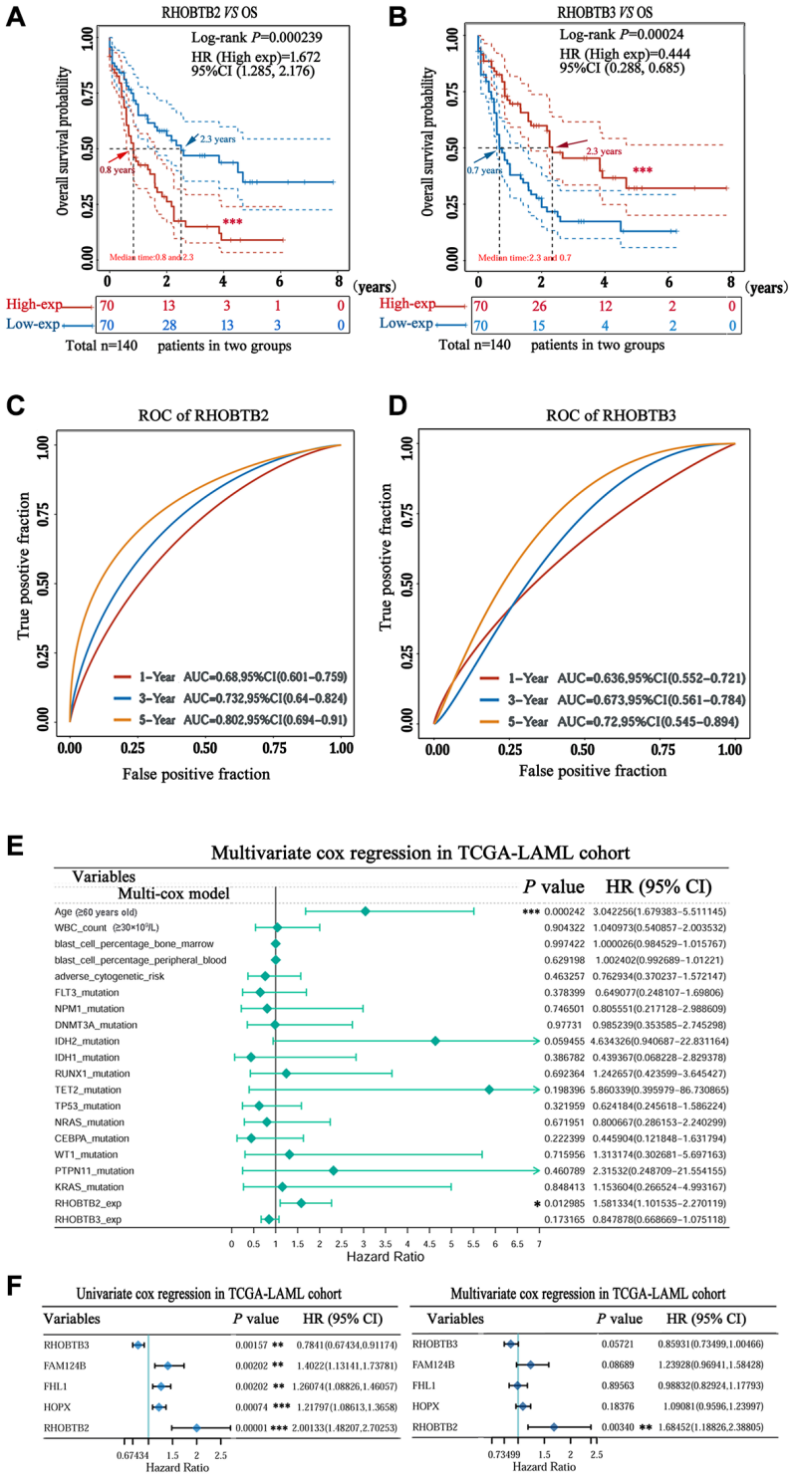
**Prognostic value of RHOBTB2 and RHOBTB3 in the TCGA-LAML cohort.** Kaplan-Meier survival analysis of RHOBTB2 (**A**) and RHOBTB3 (**B**) in AML patients. *P*-values and hazard ratios (HRs) with 95% confidence intervals (95%CIs) were generated along with log-rank tests and univariate Cox proportional hazards regression. Dotted lines indicate the 95%CI. The survival probability of a total of 140 patients from the LAML cohort was computed after case-wise deletion. Patients were grouped by a dichotomization method based on the median expression level of each gene. Solid red lines represent the high expression groups while solid blue lines represent the low expression groups. Red and blue arrows indicate the median survival time of the two groups, respectively. (**C**–**D**) Time-dependent ROC analysis was performed for the 1-, 3-, 5-year time points to determine the predictive accuracy of RHOBTB2 and RHOBTB3. AUC values represent the prediction ability in 1-, 3-, 5-year OS. ROC, receiver operating characteristic. AUC, area under the curve. (**E**) Multivariate Cox regression analyses of RHOBTB2, RHOBTB3, and clinical features in the TCGA-LAML cohort. The forest plots were generated with the *P*-values, HR, and 95% CI of each variable through ‘forestplot’ R package. (**F**) Univariate and multivariate Cox regression analyses of RHOBTB2, RHOBTB3, and three other potential prognosis-related genes (FHL1, HOPX, and FAM124B). A *P*-value < 0.05 was considered statistically significant. Asterisks represent levels of significance (^*^P < 0.05, ^**^P < 0.01, and ^***^P < 0.001).

Intriguingly, a high expression level of RHOBTB2 was associated with poor overall survival (OS) (Hazard ratio (HR) (high) = 2.9; *P* = 0.00041, Figure 2B) while a low expression level of RHOBTB3 was associated with poor OS for patients with AML (HR (high) = 0.44; *P* = 0.0045, Figure 2C) in GEPIA2. However, there was no difference for RHOBTB1 (HR (high) = 1.1; *P* = 0.85, Figure 2A). The prognostic evaluation capacity of RHOBTB2 and RHOBTB3 was validated with the UALCAN database (Supplementary Figure 2) and Kaplan-Meier (KM) survival analysis (log-rank *P*-value = 0.000239 for RHOBTB2, log-rank *P*-value = 0.00024 for RHOBTB3) in the TCGA-LAML cohort (*n* = 151) obtained from https://portal.gdc.cancer.gov/ in January 2020 (Figure 3A–3B). RHOBTB2 overexpression was recognized as a risk factor for OS with HR = 1.672 (95% confidence interval (CI), 1.285–2.176) (Figure 3A) via Cox proportional hazards analysis, while high expression of RHOBTB3 as a protective factor with HR = 0.444 (95% CI, 0.288–0.685) (Figure 3B). The median survival time of the RHOBTB2 high-expression group was 0.8 years and that of the low-expression group was 2.3 years. In comparison, the median survival times of the RHOBTB3 high and low-expression groups were 2.3 years and 0.7 years, respectively (Figure 3A–3B). Time-dependent receiver operating characteristic (ROC) analysis of RHOBTB2 and RHOBTB3 was performed to compare each gene's predictive accuracy. RHOBTB2 had a larger area under the curve (AUC) than RHOBTB3, especially for 3- and 5-year survival (3-year AUC = 0.732 *vs.* 0.673, 5-year AUC = 0.802 *vs.* 0.720) (Figure 3C–3D). Therefore, the results above suggest that both RHOBTB2 and RHOBTB3 may be potential prognostic factors for patients with leukemia, and RHOBTB2 showed better prognostic performance.

### Identification of RHOBTB2 as an independent prognostic indicator in AML

We performed univariate and multivariate Cox regression analyses to determine whether RHOBTB2 and RHOBTB3 are robust AML OS-related genes that can be used for prognosis prediction.

Multiple clinical factors, such as age, WBC count, blast cell percentage, and cytogenetic abnormalities, impact the prognosis of AML. Some individual genes, including four-and-a-half LIM domain 1(FHL1), HOPX and FAM124B have been recently identified as candidate prognostic factors through a genome-wide Cox regression screening project [17]. Thus, RHOBTB2 and RHOBTB3, combined with risk factors including age (≥60 years old), WBC count (≥30 × 10^9^/L), blast cell percentage, cytogenetic abnormalities, therapeutic agent target (FLT3, DNMT3A, and TP53 mutations, etc.), and *de novo* prognostic indicators (FHL1, HOPX, and FAM124B) (Supplementary Table 1) were used for multivariate Cox regression analysis of the TCGA-LAML cohort (*n* = 151). As shown in Figure 3E, the forest plots indicated that high RHOBTB2 expression, but not high RHOBTB3 expression, was strongly predictive of poor outcome in AML patients (HR = 1.581; 95% CI, 1.102–2.270; *P* = 0.012), independent of clinical features including age, WBC count, blast cell percentage and gene mutation status (Figure 3E). Compared to RHOBTB3 and the three potential prognostic indicators (FHL1, HOPX and FAM124B), only RHOBTB2 displayed prognostic value (*P*-value = 0.003, *vs. P*-value = 0.057 for RHOBTB3, *P*-value = 0.896 for FHL1, *P*-value = 0.087 for FAM124B, and *P*-value = 0.184 for HOPX), and a higher HR (HR = 1.685; 95% CI, 1.188–2.388) in the Cox model (Figure 3F, right panel), even though all five genes were statistically significant in univariate Cox regression analysis (Figure 3F, left panel). The results above verify that RHOBTB2 can be used as an independent and effective predictor of the OS for AML patients.

### Association of RHOBTB2 expression with AML classification and clinical characteristics

The French–American–British (FAB) classification, devised in the 1970s and 1980s, recognizes eight subtypes of AML (FAB M0-M7) based mostly on morphology and cytochemistry [18]. The more precise World Health Organization (WHO) classification has been used in recent years, which considers clinical features, morphology, immunophenotyping, cytogenetics, and molecular genetics [19]. A set of recurring chromosomal and genetic lesions related to oncogenes, tumor suppressor genes, and other regulatory elements that control vital cell functions, such as t(15;17) (q22;q12), *PML-RARα,* t(8;21) (q22;q22), *RUNX1-RUNX1T1, FLT3*-ITD mutation and *NPM1* mutation, have been introduced in many patients [19–22].

**Figure 4:**
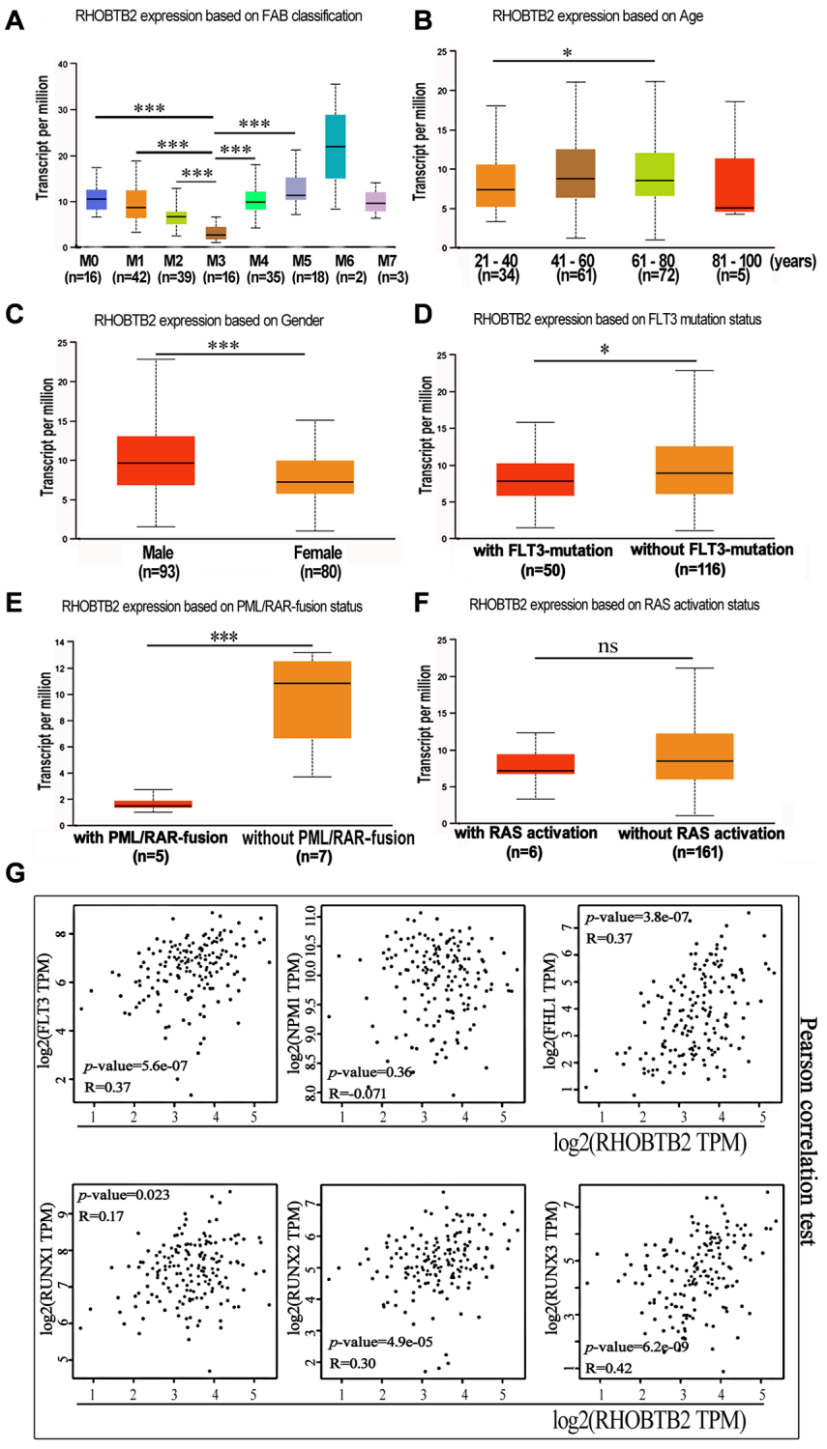
**The mRNA expression levels of RHOBTB2 in various classes of AML (UALCAN) and the correlations between RHOBTB2 and disease-related genes in the TCGA-LAML cohort (GEPIA2).** (**A**) Box plots representing RHOBTB2 expression levels in different French–American–British (FAB) subtypes. (**B**–**F**) Box plots representing RHOBTB2 expression levels in subgroups based on age (**B**), gender (**C**), FLT3 mutation (**D**), PML/RAR-fusion (**E**), and RAS activation status (**F**). (**G**) The scatter plots show the correlation between RHOBTB2 and disease-related genes such as FLT3, NPM1, FHL1, and RUNX1-3 according to Pearson’s correlation analysis (GEPIA2). A non-log scale of mRNA expression levels was used for calculation and a log2-scale axis was used for visualization. R values indicate correlation coefficients. A *P*-value < 0.05 was considered statistically significant. ^*^P < 0.05, ^**^P < 0.01, ^***^P < 0.001, ns, not significant.

We further clarified the expression profile of RHOBTB2 in different subtypes of AML using UALCAN. The box plots showed that RHOBTB2 was remarkably increased in FAB subtypes M0, M1, M2, M4, and M5 compared to that in M3 (also known as acute promyelocytic leukemia (APL)), which has a higher degree of differentiation (*P* < 0.001 for M0 *vs.* M3, M1 *vs.* M3, M2 *vs.* M3, M4 *vs.* M3 and M5 *vs.* M3, Figure 4A). FAB subtypes M6 and M7 were not considered because of the small sample size for each. It seems like progenitor cells have higher RHOBTB2 expression levels than their progeny.

Next, the correlation between RHOBTB2 expression and clinical features, such as age, gender, FLT3 mutation, PML/RAR-fusion, and RAS activation status in AML patients, was analyzed through UALCAN (Figure 4B–4F). Box plots showed that the expression level of RHOBTB2 was higher in the 61–80-year-old group than in the 21–40-year-old group (*P* = 0.015, Figure 4B) and higher in the male group than in the female group (*P* < 0.001, Figure 4C). We did not analyze the 80–100-year-old group, as it had only five samples. In addition, RHOBTB2 expression was increased in AML patients without FLT3 mutation (*P* = 0.024, Figure 4D) and patients without PML-RAR fusion (*P* = 0.0038, Figure 4E). The presence of FLT3 mutations in AML enabled the recent approval of targeted drugs that can help patients achieve prolonged remission. PML-RAR is a fusion gene that is associated with the specific subtype of leukemia APL.

Furthermore, the correlation between RHOBTB2 and disease-related genes was analyzed. The statistical scatter plots from the GEPIA2 database showed that RHOBTB2 expression had a positive association with the expression of FLT3 (Pearson’s correlation = 0.37, *P* = 5.56E-07), FHL1 (Pearson’s correlation = 0.37, *P* = 3.8E-07), RUNX1 (Pearson’s correlation = 0.17, *P* = 0.023), RUNX2 (Pearson’s correlation = 0.3, *P* = 4.9E-05), and RUNX3 (Pearson’s correlation = 0.42, *P* = 6.2E-09) (Figure 4G). FLT3 is overexpressed on CD34^+^ blast cells in approximately 93% of AML cases and might share the same expression pattern with RHOBTB2 in bone marrow samples. High expression of FHL1 and RUNX1-3 is related to poor prognosis. NPM1, the high expression of which predicts a superior prognosis, was negatively correlated with RHOBTB2, but the correlation was not significant.

### Functional enrichment analyses of RHOBTB2 and co-expressed genes in AML

**Figure 5:**
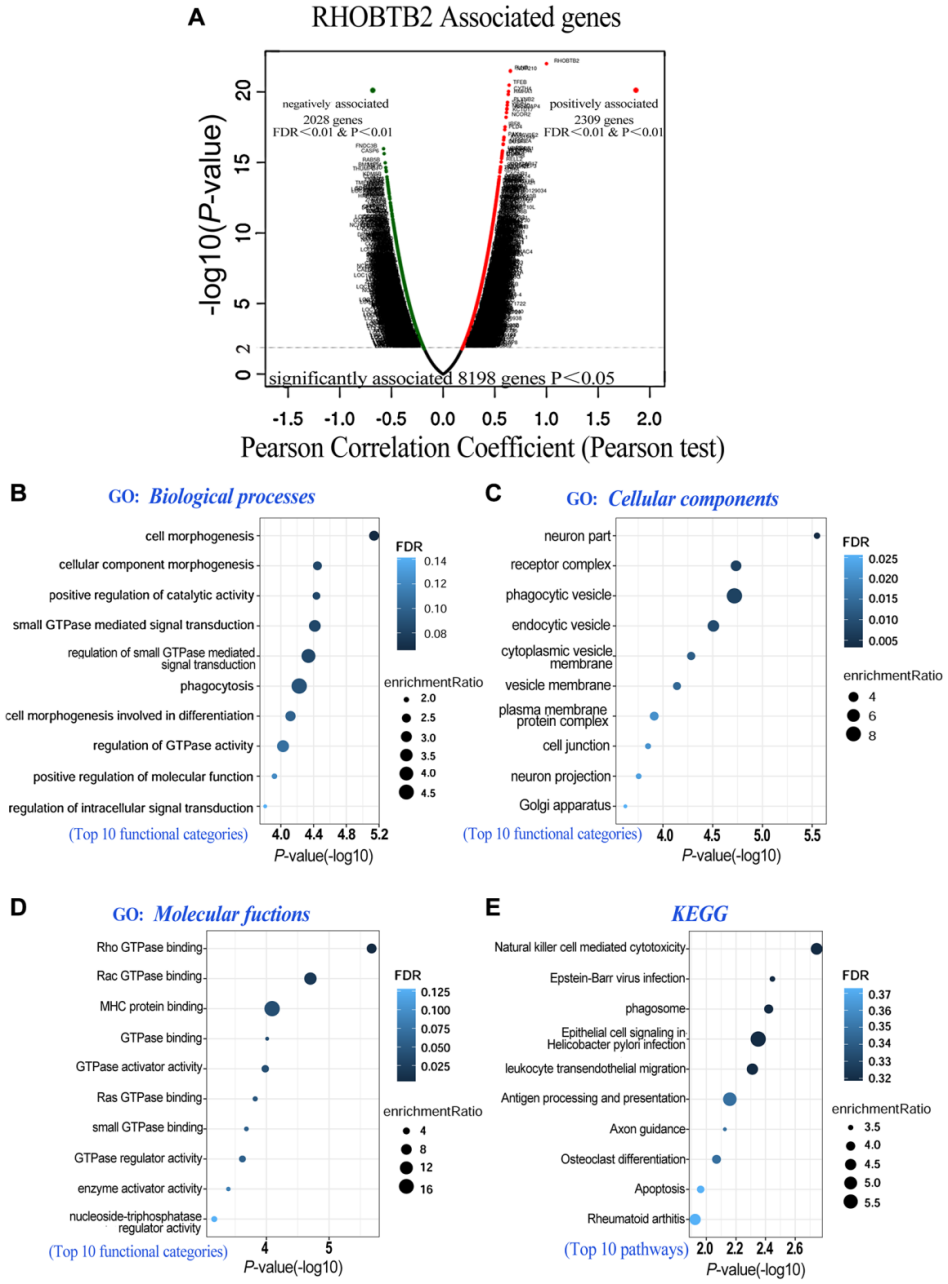
**Co-expressed genes of RHOBTB2 (LinkedOmics) and functional enrichment analyses in AML (WebGestalt).** (**A**) Genes positively and negatively correlated with RHOBTB2 in AML were indicated by the volcano plot. Red dots in the right sector represent positively correlated genes, while green dots in the left sector are negatively correlated genes. A total of 8,198 genes with significant associations were defined (*P* < 0.05), among which there were 2,309 positively associated genes and 2,028 negatively associated genes when FDR < 0.01 and *P*-value < 0.01 were used as thresholds. Pearson’s test was used to identify the correlations in the TCGA-LAML cohort (*n* = 173). Bubble plots display the functional enrichment results of GO analysis in terms of biological processes (**B**), cellular components (**C**), molecular functions (**D**) and KEGG signaling pathways (**E**). The top 10 functional categories and pathways were annotated with color gradient bubbles of different sizes. A (-log10) *P*-value>1.3 (*P*-value < 0.05) was considered statistically significant. FDR, false discovery rate.

To further explore the potential function and molecular pathways of the RHOBTB2 gene in AML, we utilized the LinkedOmics database [23] to identify co-expressed genes of RHOBTB2 in data of 173 patients from TCGA. A total of 8,198 genes related to RHOBTB2 were altered, which reflects the considerable impact of the core gene RHOBTB2 on AML pathogenesis. The 2,309 gene clusters of these related genes that were positively related to RHOBTB2 are displayed as red dots, whereas the 2,028 gene clusters that were negatively associated with RHOBTB2 are represented by green dots in the volcano plot (*P* < 0.01 and FDR < 0.01, Figure 5A). The top 20 significant gene sets positively and negatively associated with RHOBTB2 are presented in Table 1.

**Table 1:** Representative genes positively and negatively associated to RHOBTB2 by Pearson’s test from LinkedOmics database.

**Associated genes**	**Pearson Correlation Coefficient**	***P*-value**	**FDR (BH)**
*Top 20 positively associated genes*			
FLNB	0.650972985	3.16E-22	2.24E-18
NUP210	0.650504829	3.46E-22	2.24E-18
TFEB	0.638588116	3.33E-21	1.62E-17
CYTH4	0.632993672	9.32E-21	3.63E-17
HMHA1	0.630347457	1.51E-20	4.88E-17
PLXNB2	0.623049163	5.52E-20	1.53E-16
SIPA1	0.620465063	8.68E-20	2.11E-16
MEF2D	0.61724265	1.52E-19	3.28E-16
ARHGAP4	0.616523644	1.72E-19	3.34E-16
KCTD17	0.613370759	2.94E-19	5.19E-16
NCOR2	0.608853961	6.28E-19	1.02E-15
IRF8	0.599255299	3.04E-18	4.55E-15
PLD4	0.596469425	4.76E-18	6.61E-15
PAK1	0.588692953	1.62E-17	2.10E-14
ATP6V0E2	0.587904178	1.84E-17	2.23E-14
KIAA1949	0.585777691	2.55E-17	2.92E-14
MFSD2A	0.581547428	4.87E-17	5.26E-14
DUSP7	0.580947776	5.34E-17	5.46E-14
LILRB4	0.573953331	1.52E-16	1.41E-13
UNC93B1	0.573436959	1.64E-16	1.45E-13
*Top 20 negatively associated genes*			
FNDC3B	–0.576323492	1.07E-16	1.04E-13
CASP6	–0.570890084	2.39E-16	1.86E-13
RAB5B	–0.560513869	1.06E-15	6.47E-13
MST4	–0.554995474	2.31E-15	1.25E-12
BMS1P5	–0.554977234	2.31E-15	1.25E-12
BLID	–0.551717218	3.63E-15	1.81E-12
THUMPD1	–0.550233952	4.45E-15	2.11E-12
KDM5B	–0.543785876	1.06E-14	4.70E-12
CLINT1	–0.540830204	1.58E-14	6.56E-12
SIX3	–0.538128546	2.25E-14	8.80E-12
ITPR2	–0.537446438	2.46E-14	9.21E-12
FAM5B	–0.536216849	2.89E-14	1.04E-11
MAX	–0.534375272	3.67E-14	1.19E-11
AVEN	–0.534276355	3.72E-14	1.19E-11
TMEM87A	–0.532895715	4.45E-14	1.37E-11
TRNT1	–0.531726008	5.17E-14	1.55E-11
RPGR	–0.531267221	5.49E-14	1.62E-11
APLF	–0.529254158	7.10E-14	2.01E-11
PIK3CB	–0.528410674	7.90E-14	2.13E-11
TM7SF3	–0.527464794	8.91E-14	2.34E-11

FDR(BH): False discovery rate is calculated by BH (Benjamini-Hochberg method).

RHOBTB2 and its top 200 associated gene clusters were subjected to gene ontology (GO) and Kyoto Encyclopedia of Genes and Genomes (KEGG) functional enrichment analyses to identify enriched categories and signaling pathways in the TCGA-LAML cohort. The bubble diagrams showed that the gene clusters were located in cell morphogenesis involved in differentiation, small GTPase mediated signal transduction, and positive regulation of catalytic activity (http://amigo.geneontology.org/amigo, GO:0000902, GO:0000904, GO:0007264, GO:0043085, etc) in the aspect of biological processes (Figure 5B). The functions of these categories above may be related to cell division, cell cycle, proliferation, and differentiation of blast cells in AML. In the aspect of cellular components, Chang et al. found that RhoBTB2 is distributed in a vesicular pattern [24], and coincidentally these genes are putative structural constituents of phagocytic vesicle (GO:0045335), endocytic vesicle (GO:0030139) and cytoplasmic vesicle membrane (GO:0030659) (Figure 5C). When it comes to the aspect of molecular functions, they are localized in RHOBTB2-related utilities such as Rho GTPase binding (GO:0017048), Rac GTPase binding (GO:0048365) and GTPase activator activity (GO:0005096) (Figure 5D). The top 10 pathways related to RHOBTB2 and co-expressed gene clusters were defined by KEGG analysis. These pathways such as cytotoxicity (https://www.kegg.jp/kegg/, hsa04650), phagosome (hsa04145), leukocyte transendothelial migration (hsa04670), and apoptosis (hsa04210), have been intriguingly implicated in vital pathological processes including cell fate determination and migration of leukocytes (Figure 5E). Given our results and the architecture, localization and biological functions of RHOBTB2 postulated in previous research, we hypothesized that overexpression of RHOBTB2 in leukemic blast cells could regulate cell differentiation, cell cycle, proliferation, apoptosis, migration and vesicle transport.

## MATERIALS AND METHODS

### GEPIA2

GEPIA2 (http://gepia2.cancer-pku.cn/, Beijing, China) is an interactive web-based tool for analyzing cancer-related RNA sequencing data provided by TCGA and GTEx projects [25]. General gene expression profiles, survival analysis, and correlation analysis were conducted through the “Expression Analysis” module with the TCGA- LAML cohort (*n* = 173) and normal tissues (*n* = 70), the data of which are available in the panel “dataset sources”. Student’s *t*-test was used to perform expression analysis. The survival results were displayed by Kaplan-Meier curves with HRs and *P* values from a log-rank test. A *P*-value = 0.05 was used as the threshold of statistical significance.

### Univariate and multivariate Cox regression analyses

Univariate and multivariate Cox regression analyses were performed to identify candidate prognostic genes in the LAML cohort from TCGA. The data (available through https://portal.gdc.cancer.gov/) were updated in Jan 2020, and the cohort contains 151 AML patients with high-throughput sequencing (RNA-Seq) data and detailed clinical information [26].

A forest plot with the *P*-value, HR and 95% CI of each variable was built through “survival”, “survminer” and “forestplot” R packages in RStudio 4.0.3. Gene expression levels were dichotomized based on the median expression level in the cohort as the cutoff value.

### LinkedOmics

LinkedOmics (http://www.linkedomics.org) provides a unique portal to analyze cancer multi-omics data and clinical data for 32 cancer types and 11 158 patients from TCGA project [23]. Genes associated with RHOBTB2 were identified in the TCGA-LAML cohort (*n* = 173) and are presented in volcano plots. Pearson’s correlation test was used to evaluate the statistical relationship.

### UALCAN

UALCAN (Birmingham, AL, USA, http://ualcan.path.uab.edu) serves as a platform for validating specific genes and screening tumor candidate biomarkers [27]. RHOBTB gene expression in AML subgroups based on various clinicopathologic features and survival outcomes was investigated via “Expression Analysis” and “Survival Analysis” modules, respectively. The processed RNA-sequencing data and survival profiles of the AML cohort (*n* = 163) were obtained using TCGA assembler (http://www.compgenome.org/TCGA-Assembler/). A *P-*value of 0.05 was used as the threshold for significance.

### GO and KEGG pathway enrichment analyses

The WebGestalt database (http://www.webgestalt.org/option.php) for deriving biological insights from gene lists was exploited to perform GO and KEGG pathway enrichment analyses for RHOBTB2 and the top 200 co-expressed genes. The built-in reference human protein-coding genome was selected as the background parameter. Bubble plots with (-log10) *P-*values, FDRs, and enrichment ratios were generated through “ggplot” and “dplyr” R packages. A (-log10) *P-*value > 1.3 was considered to indicate enrichment of a meaningful pathway.

## DISCUSSION

As an atypical subfamily of Rho GTPases, RhoBTB proteins possess the most salient domain architecture. Studies have implicated their pivotal role in the regulation of cell growth through cell cycle control and apoptosis, vesicle trafficking, and organization of the actin filament system. Hitherto increasing evidence has implicated the RHOBTB genes in tumorigenesis. The expression profile of RHOBTB genes in AML and whether they can affect myeloid leukemogenesis, pathogenesis, and prognosis remain obscure. In this study, we performed bioinformatics analyses to explore the expression profile and prognostic value of RHOBTB genes in AML and enhance the accuracy of prognosis prediction.

We found aberrant RHOBTB gene expression in human AML samples through the ONCOMINE and GEPIA2 databases. RHOBTB1 and RHOBTB3 were decreased significantly in AML samples. In contrast, the transcriptional level of RHOBTB2 was dramatically increased in AML compared to normal samples, unlike the pattern found in other tumors. All three RHOBTB genes have notable differences in tissue expression levels in humans [6]. The status of RHOBTB genes in various tumors remains to be further uncovered. Although RHOBTB2 is frequently deleted in various carcinomas, including breast, lung, and stomach carcinomas, many tumor cells still retain RHOBTB2 expression [28]. Blast cells make up a high proportion (20%~100%) of the cells in bone marrow samples from AML patients. These myeloid progenitor cells are derived from hematopoietic stem cells (alias leukemia stem cells (LSCs)), which are different from the stem cells of solid tumors. Based on the findings above, we hypothesized that there is no overt relationship between mRNA expression patterns of the three RHOBTB genes and protein architecture. It is reasonable that only RHOBTB2 showed expression patterns in AML patients that are different from those in solid tumor types.

The prognostic value of RHOBTB genes in patients with AML was assessed in several databases and by Cox regression. Survival analysis suggested that high RHOBTB2 expression and low RHOBTB3 expression are associated with adverse OS in AML. The ROC analysis indicated that RHOBTB2 had a larger AUC than RHOBTB3 and had a better prognostic value. Subsequently, to demonstrate whether the prognostic efficacy of RHOBTB2 and RHOBTB3 is independent of other clinical factors, we performed multivariate Cox regression analyses in the TCGA-AML cohort. Several disease-related factors and gene mutations, such as age, WBC count, blast cell percentage, TP53 mutations, FHL1, HOPX, and FAM124B, were confirmed to have significant and general prognostic value in previous studies [17]. We entered RHOBTB2 and RHOBTB3 with all of these prognostic variables into the multivariate analyses. High RHOBTB2 expression was identified as an independent indicator for unfavorable OS.

We examined the relationship between RHOBTB2 expression and clinical features and genetic alterations of AML patients to validate whether it could be used as a tool for risk stratification. RHOBTB2 expression was higher in the 61–80-year-old group, which is consistent with the worse 5-year OS of elderly AML patients. The RHOBTB2 expression level was upregulated in the non-APL FAB subtype, AML patients without FLT3 mutation, and patients without PML-RAR fusion, although it showed no difference between patients with and without RAS activation status. Correlation analysis with the GEPIA2 database indicated that the RHOBTB2 expression was positively associated with the expression of FLT3, FHL1, and RUNX1-3. Patients with FLT3 mutation have a lower complete remission rate and poorer prognosis [29]. FHL1 is a powerful prognostic factor for determining OS, event-free survival, and relapse-free survival [17]. The three RUNX family members are lineage-specific master regulators and play an essential role in hematopoiesis [30]. These data reinforce the role of RHOBTB2 as a prognostic indicator for specific AML subtypes.

We also explored the potential of RHOBTB2 co-expressed genes as biomarkers for AML through the LinkedOmics database. GO and KEGG analyses indicated that these co-expressed genes were enriched in multiple functional categories and pathways that may contribute to regulating the cell cycle, apoptosis, differentiation, migration and vesicle transport in AML. Although there is little research concerning the role of RHOBTB2 in AML, in future studies, we will aim to determine the possible mechanism based on the comprehensive analysis of the expression patterns and functional enrichment in AML. RhoBTB2 can function in cell cycle and apoptosis through ubiquitination and degradation of cancer-related proteins, so we hypothesize that RhoBTB2 plays an intricate and differential role in tumorigenesis depending on target genes with multiple pathways. RHOBTB2 (DBC2) downregulates cyclin D1 (CCND1); however, other leukemogenesis pathways, such as c-myc and Wint-1, might be induced [28]. Scott N. Freeman et al. found that RHOBTB2 overexpression, as a target of E2F1 during mitosis, facilitates cell cycle progression and propagation for a short time [31]. E2F1 may promote the transcription of RHOBTB2 during mitosis, which affects the cell cycle and boosts the proliferation of AML leukemia cells. RHOBTB2 is expressed in fetal tissues and may control developmental processes [32], thus possibly exerting an influence on morphogenesis, localization and differentiation of leukemic blast cells. The potential role of RhoBTB2 in vesicle transport has been addressed by Chang et al. [24]. We hypothesized that RhoBTB2 might partly mediate the membrane trafficking and distribution of chemotherapeutic drugs and thus contribute to the poor outcomes of AML treatment, but the in-depth mechanism requires more laboratory work. The published reports above have introduced some intriguing hypotheses that provide the basis for further explorations.

In conclusion, the current study was the first to thoroughly identify the aberrant expression and prognostic value of RHOBTB family members in AML. RHOBTB2 was increased in high-risk subgroups of patients with leukemia and thus could serve as a potential biomarker. Our results illustrate that the overexpression of RHOBTB2 is an independent indicator for predicting the adverse outcome of AML and might play an essential role in leukemogenesis. Further research based on this discovery will aid the understanding of the comprehensive gene network of leukemogenesis and improve the accuracy of leukemia survival and prognostic prediction.

## Supplementary Materials

Supplementary Figures

Supplementary Table 1

## References

[1] Siegel RL, Miller KD, Jemal A. Cancer statistics, 2015. CA Cancer J Clin. 2015; 65:5–29. 10.3322/caac.21254 25559415

[2] Aldoss I, Marcucci G. More options for older patients with acute myeloid leukemia: venetoclax in combination with low dose cytarabine. Chin Clin Oncol. 2019; 8:S25. 10.21037/cco.2019.09.03 31684734

[3] Grove CS, Vassiliou GS. Acute myeloid leukaemia: a paradigm for the clonal evolution of cancer? Dis Model Mech. 2014; 7:941–51. 10.1242/dmm.015974 25056697 PMC4107323

[4] Meyer SC, Levine RL. Translational implications of somatic genomics in acute myeloid leukaemia. Lancet Oncol. 2014; 15:e382–94. 10.1016/S1470-2045(14)70008-7 25079101

[5] Papaemmanuil E, Gerstung M, Bullinger L, Gaidzik VI, Paschka P, Roberts ND, Potter NE, Heuser M, Thol F, Bolli N, Gundem G, Van Loo P, Martincorena I, et al. Genomic Classification and Prognosis in Acute Myeloid Leukemia. N Engl J Med. 2016; 374:2209–21. 10.1056/NEJMoa1516192 27276561 PMC4979995

[6] Ramos S, Khademi F, Somesh BP, Rivero F. Genomic organization and expression profile of the small GTPases of the RhoBTB family in human and mouse. Gene. 2002; 298:147–57. 10.1016/s0378-1119(02)00980-0 12426103

[7] Aspenström P, Ruusala A, Pacholsky D. Taking Rho GTPases to the next level: the cellular functions of atypical Rho GTPases. Exp Cell Res. 2007; 313:3673–79. 10.1016/j.yexcr.2007.07.022 17850788

[8] Hamaguchi M, Meth JL, von Klitzing C, Wei W, Esposito D, Rodgers L, Walsh T, Welcsh P, King MC, Wigler MH. DBC2, a candidate for a tumor suppressor gene involved in breast cancer. Proc Natl Acad Sci U S A. 2002; 99:13647–52. 10.1073/pnas.212516099 12370419 PMC129730

[9] Mao H, Qu X, Yang Y, Zuo W, Bi Y, Zhou C, Yin H, Deng B, Sun J, Zhang L. A novel tumor suppressor gene RhoBTB2 (DBC2): frequent loss of expression in sporadic breast cancer. Mol Carcinog. 2010; 49:283–89. 10.1002/mc.20598 19937980

[10] Dong W, Meng L, Shen HC, Du JJ. Loss of DBC2 expression is an early and progressive event in the development of lung adenocarcinoma. Asian Pac J Cancer Prev. 2012; 13:2021–23. 10.7314/apjcp.2012.13.5.2021 22901165

[11] Knowles MA, Aveyard JS, Taylor CF, Harnden P, Bass S. Mutation analysis of the 8p candidate tumour suppressor genes DBC2 (RHOBTB2) and LZTS1 in bladder cancer. Cancer Lett. 2005; 225:121–30. 10.1016/j.canlet.2004.10.047 15922864

[12] Shi Y, Chen JY, Yang J, Li B, Chen ZH, Xiao CG. DBC2 gene is silenced by promoter methylation in bladder cancer. Urol Oncol. 2008; 26:465–69. 10.1016/j.urolonc.2007.08.009 18640857

[13] Jin Z, Han YX, Han XR. Downregulated RhoBTB2 expression contributes to poor outcome in osteosarcoma patients. Cancer Biother Radiopharm. 2013; 28:709–16. 10.1089/cbr.2012.1386 23777252

[14] Cho YG, Choi BJ, Kim CJ, Song JH, Zhang C, Nam SW, Lee JY, Park WS. Genetic analysis of the DBC2 gene in gastric cancer. Acta Oncol. 2008; 47:366–71. 10.1080/02841860701644094 17906984

[15] Xu RS, Wu XD, Zhang SQ, Li CF, Yang L, Li DD, Zhang BG, Zhang Y, Jin JP, Zhang B. The tumor suppressor gene RhoBTB1 is a novel target of miR-31 in human colon cancer. Int J Oncol. 2013; 42:676–82. 10.3892/ijo.2012.1746 23258531

[16] Zhang CS, Liu Q, Li M, Lin SY, Peng Y, Wu D, Li TY, Fu Q, Jia W, Wang X, Ma T, Zong Y, Cui J, et al. RHOBTB3 promotes proteasomal degradation of HIFα through facilitating hydroxylation and suppresses the Warburg effect. Cell Res. 2015; 25:1025–42. 10.1038/cr.2015.90 26215701 PMC4559813

[17] Fu Y, Xu M, Cui Z, Yang Z, Zhang Z, Yin X, Huang X, Zhou M, Wang X, Chen C. Genome-wide identification of FHL1 as a powerful prognostic candidate and potential therapeutic target in acute myeloid leukaemia. EBioMedicine. 2020; 52:102664. 10.1016/j.ebiom.2020.102664 32062360 PMC7021551

[18] Goldman SL, Hassan C, Khunte M, Soldatenko A, Jong Y, Afshinnekoo E, Mason CE. Epigenetic Modifications in Acute Myeloid Leukemia: Prognosis, Treatment, and Heterogeneity. Front Genet. 2019; 10:133. 10.3389/fgene.2019.00133 30881380 PMC6405641

[19] Vardiman JW, Thiele J, Arber DA, Brunning RD, Borowitz MJ, Porwit A, Harris NL, Le Beau MM, Hellström-Lindberg E, Tefferi A, Bloomfield CD. The 2008 revision of the World Health Organization (WHO) classification of myeloid neoplasms and acute leukemia: rationale and important changes. Blood. 2009; 114:937–51. 10.1182/blood-2009-03-209262 19357394

[20] Estey E. Acute myeloid leukemia: 2016 Update on risk-stratification and management. Am J Hematol. 2016; 91:824–46. 10.1002/ajh.24439 27417880

[21] Estey EH. Acute myeloid leukemia: 2019 update on risk-stratification and management. Am J Hematol. 2018; 93:1267–91. 10.1002/ajh.25214 30328165

[22] Estey EH. Acute myeloid leukemia: 2021 update on risk-stratification and management. Am J Hematol. 2020; 95:1368–98. 10.1002/ajh.25975 32833263

[23] Vasaikar SV, Straub P, Wang J, Zhang B. LinkedOmics: analyzing multi-omics data within and across 32 cancer types. Nucleic Acids Res. 2018; 46:D956–63. 10.1093/nar/gkx1090 29136207 PMC5753188

[24] Chang FK, Sato N, Kobayashi-Simorowski N, Yoshihara T, Meth JL, Hamaguchi M. DBC2 is essential for transporting vesicular stomatitis virus glycoprotein. J Mol Biol. 2006; 364:302–08. 10.1016/j.jmb.2006.09.026 17023000 PMC1713265

[25] Tang Z, Kang B, Li C, Chen T, Zhang Z. GEPIA2: an enhanced web server for large-scale expression profiling and interactive analysis. Nucleic Acids Res. 2019; 47:W556–60. 10.1093/nar/gkz430 31114875 PMC6602440

[26] Ley TJ, Miller C, Ding L, Raphael BJ, Mungall AJ, Robertson A, Hoadley K, Triche TJ Jr, Laird PW, Baty JD, Fulton LL, Fulton R, Heath SE, et al, and Cancer Genome Atlas Research Network. Genomic and epigenomic landscapes of adult de novo acute myeloid leukemia. N Engl J Med. 2013; 368:2059–74. 10.1056/NEJMoa1301689 23634996 PMC3767041

[27] Chandrashekar DS, Bashel B, Balasubramanya SAH, Creighton CJ, Ponce-Rodriguez I, Chakravarthi BV, Varambally S. UALCAN: A Portal for Facilitating Tumor Subgroup Gene Expression and Survival Analyses. Neoplasia. 2017; 19:649–58. 10.1016/j.neo.2017.05.002 28732212 PMC5516091

[28] Yoshihara T, Collado D, Hamaguchi M. Cyclin D1 down-regulation is essential for DBC2's tumor suppressor function. Biochem Biophys Res Commun. 2007; 358:1076–79. 10.1016/j.bbrc.2007.05.037 17517369 PMC1934618

[29] Port M, Böttcher M, Thol F, Ganser A, Schlenk R, Wasem J, Neumann A, Pouryamout L. Prognostic significance of FLT3 internal tandem duplication, nucleophosmin 1, and CEBPA gene mutations for acute myeloid leukemia patients with normal karyotype and younger than 60 years: a systematic review and meta-analysis. Ann Hematol. 2014; 93:1279–86. 10.1007/s00277-014-2072-6 24801015

[30] Ito Y, Bae SC, Chuang LS. The RUNX family: developmental regulators in cancer. Nat Rev Cancer. 2015; 15:81–95. 10.1038/nrc3877 25592647

[31] Freeman SN, Ma Y, Cress WD. RhoBTB2 (DBC2) is a mitotic E2F1 target gene with a novel role in apoptosis. J Biol Chem. 2008; 283:2353–62. 10.1074/jbc.M705986200 18039672 PMC2268526

[32] St-Pierre B, Jiang Z, Egan SE, Zacksenhaus E. High expression during neurogenesis but not mammogenesis of a murine homologue of the Deleted in Breast Cancer2/Rhobtb2 tumor suppressor. Gene Expr Patterns. 2004; 5:245–51. 10.1016/j.modgep.2004.07.009 15567721

