# Supplementary material for: Identification of RHOBTB2 aberration as an independent prognostic indicator in acute myeloid leukemia
Source: Aging (Albany NY). 2021 Jun 1;13(11):15269–84. doi: 10.18632/aging.203087 (PMC8221349; doi:10.18632/aging.203087)
Supplement: Supplementary Figures [file aging-13-203087-s001.pdf]

SUPPLEMENTARY FIGURES

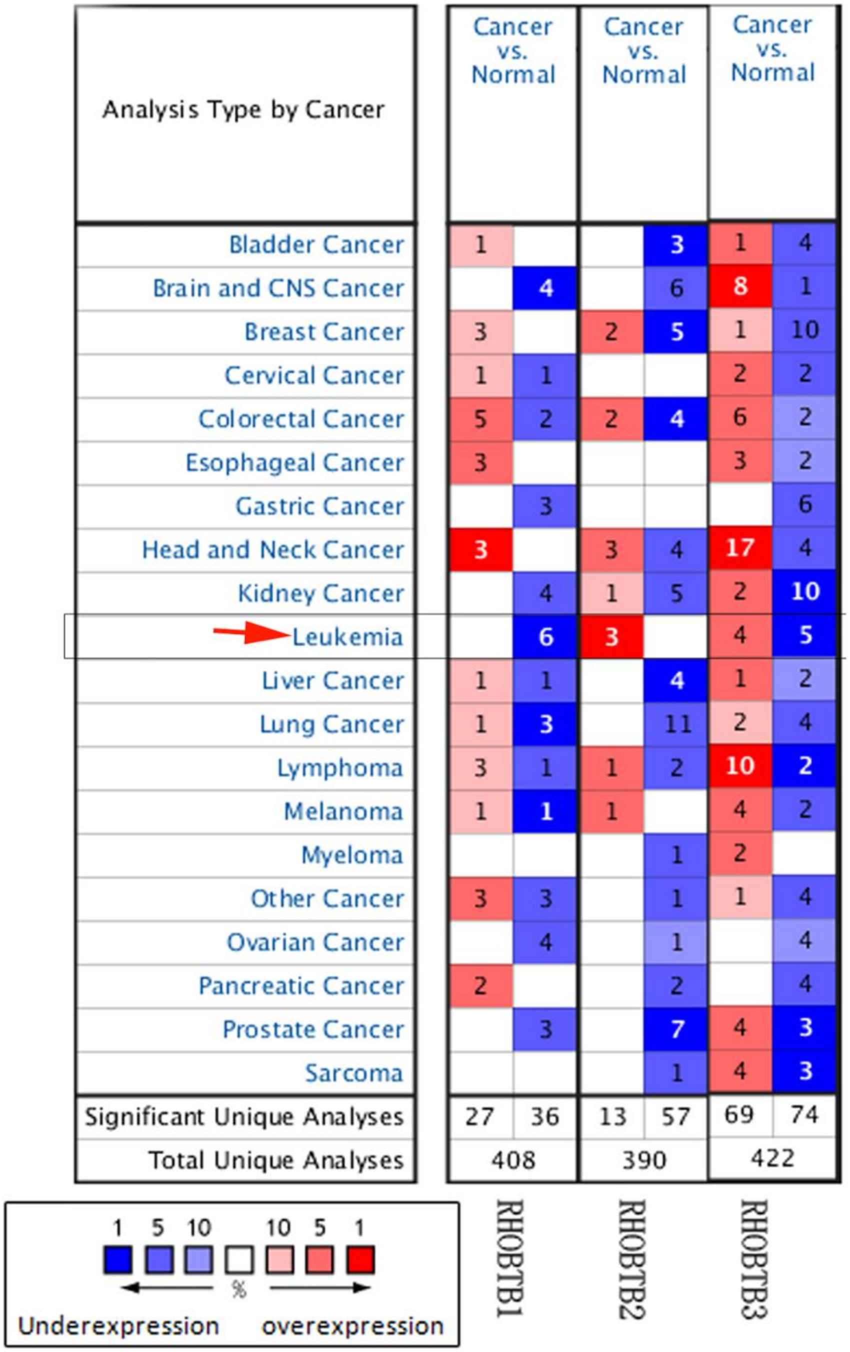

Supplementary Figure 1. RHOBTB family members transcription levels in human cancers. The mRNA expression of RHOBTB 1-3 (cancer *vs*. normal tissue) in pan-cancers was analyzed through the ONCOMINE database. The red arrow indicates RHOBTB expression in AML. The number in the colored cell represents the number of datasets meeting thresholds ($P < 0.05$, fold change = ALL). The cell color was defined as the gene rank percentile in the study.

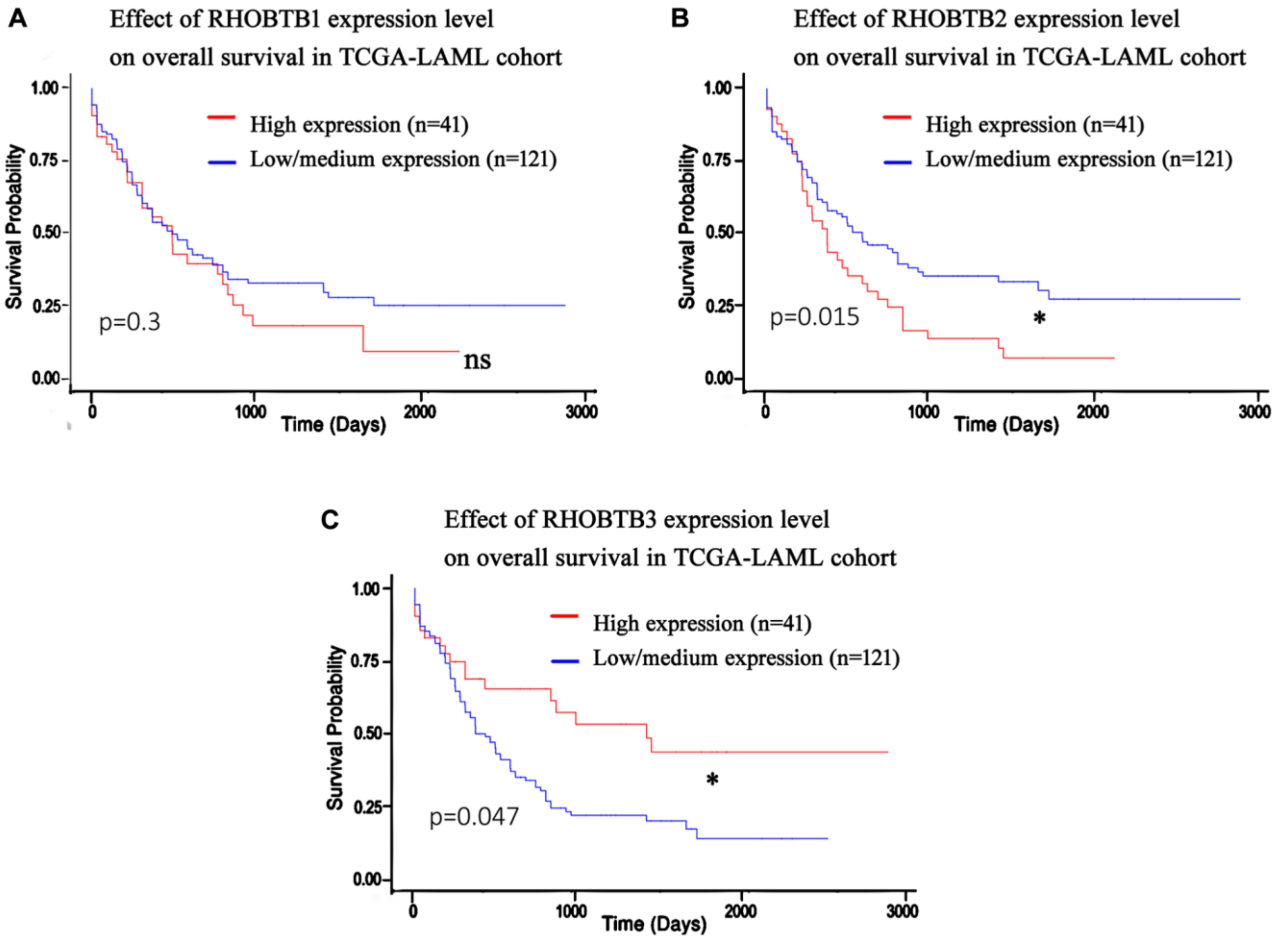

Supplementary Figure 2. The effect of RHOBTB genes expression level on overall survival of AML patients (UALCAN). The overall survival (OS) curves of AML patients in different RHOBTB1 (**A**), RHOBTB2 (**B**), and RHOBTB3 (**C**) expression groups were drawn by Kaplan-Meier survival analysis. Patients in the TCGA-LAML cohort were divided into a high expression group (n = 41) and a low/medium expression group (n = 121) according to the average expression level of each gene. $^{*}P < 0.05$.
