# Supplementary material for: Identification of RHOBTB2 aberration as an independent prognostic indicator in acute myeloid leukemia
Source: Aging (Albany NY). 2021 Jun 1;13(11):15269–84. doi: 10.18632/aging.203087 (PMC8221349; doi:10.18632/aging.203087)
Supplement: Supplementary Table 1 [file aging-13-203087-s002.pdf]

SUPPLEMENTARY TABLE

Supplementary Table 1. Univariate cox regression of clinicopathological characteristics and selected features with overall survival in a TCGA-LAML cohort.

Characteristics	HR (95% CI)	*p* value
Age (≥60 years old)	2.947 (1.998–4.348)	5.083e-08***
WBC count (≥30 × 10^9/L)	1.052 (0.718–1.542)	0.794
blast cell percentage (bone_marrow)	1.003 (0.995–1.011)	0.444
blast cell percentage (peripheral_blood)	1.001 (0.995–1.007)	0.796
RHOBTB2_exp	1.672 (1.285–2.176)	0.0001***
RHOBTB3_exp	0.741 (0.637–0.862)	0.0001***
adverse cytogenetic risk	1.622 (1.022–2.573)	0.040*
FLT3_mutation	1.958 (0.925–4.143)	0.079
NPM1_mutation	0.752 (0.325–1.739)	0.505
DNMT3A_mutation	0.938 (0.496–1.774)	0.845
IDH2_mutation	1.403 (0.565–3.480)	0.465
IDH1_mutation	1.056 (0.426–2.613)	0.907
RUNX1_mutation	0.926 (0.462–1.854)	0.828
TET2_mutation	1.542 (0.376–6.330)	0.548
TP53_mutation	2.343 (1.193–4.602)	0.013*
NRAS_mutation	0.618 (0.268–1.422)	0.258
CEBPA_mutation	0.756 (0.237–2.413)	0.636
WT1_mutation	1.801 (0.568–5.714)	0.318
PTPN11_mutation	1.202 (0.295–4.903)	0.797
KRAS_mutation	1.413 (0.515–3.874)	0.502

$^{*}P < 0.05$. $^{***}P < 0.001$.
